# Mastering Surface Sulfidation of MnP‐MnO_2_ Heterostructure to Facilitate Efficient Polysulfide Conversion in Li─S Batteries

**DOI:** 10.1002/advs.202403391

**Published:** 2024-06-24

**Authors:** Fengxing Liang, Qiao Deng, Shunyan Ning, Huibing He, Nannan Wang, Yanqiu Zhu, Jinliang Zhu

**Affiliations:** ^1^ School of Resources Environment and Materials State Key Laboratory of Featured Metal Materials and Life‐cycle Safety for Composite Structures Guangxi University Nanning 530004 P. R. China; ^2^ School of Nuclear Science and Technology University of South China 28 Changsheng West Road Hengyang 421001 P. R. China; ^3^ Faculty of Environment, Science and Economy University of Exeter Exeter EX44QF United Kingdom

**Keywords:** heterostructure, in situ characterization, lithium–sulfur batteries, manganese phosphide, surface sulfidation

## Abstract

The development of lithium–sulfur (Li─S) batteries has been hampered by the shuttling effect of lithium polysulfides (LiPSs). An effective method to address this issue is to use an electrocatalyst to accelerate the catalytic conversion of LiPSs. In this study, heterogeneous MnP‐MnO_2_ nanoparticles are uniformly synthesized and embedded in porous carbon (MnP‐MnO_2_/C) as core catalysts to improve the reaction kinetics of LiPSs. In situ characterization and density functional theory (DFT) calculations confirm that the MnP‐MnO_2_ heterostructure undergo surface sulfidation during the charge/discharge process, forming the MnS_2_ phase. Surface sulfidation of the MnP‐MnO_2_ heterostructure catalyst significantly accelerated the SRR and Li_2_S activation, effectively inhibiting the LiPSs shuttling effect. Consequently, the MnP‐MnO_2_/C@S cathode achieves outstanding rate performance (10 C, 500 mAh g^−1^) and ultrahigh cycling stability (0.017% decay rate per cycle for 2000 cycles at 5 C). A pouch cell with MnP‐MnO_2_/C@S cathode delivers a high energy density of 429 Wh kg^−1^. This study may provide a new approach to investigating the surface sulfidation of electrocatalysts, which is valuable for advancing high‐energy‐density Li−S batteries.

## Graphical abstract

In an aprotic environment rich in lithium polysulfides, the MnP‐MnO_2_ heterostructure catalyst will undergo surface sulfidation, forming the MnS_2_ phase. The formation of the MnS_2_ phase primarily accelerates the sulfur reduction reaction (SRR) kinetics, effectively inhibiting the LiPSs shuttling effect. Benefiting from this, the Li─S pouch cell exhibited a high initial energy density of 429 Wh kg^−1^.

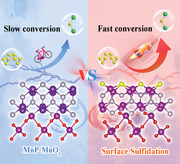

## 1 Introduction

Lithium–sulfur (Li─S) batteries are promising next‐generation energy storage technologies owing to their ultrahigh theoretical energy density, cost‐effectiveness, and environmental friendliness.^[^ ^1^ ^]^ Unfortunately, the sluggish conversion kinetics of LiPSs lead to their continuous accumulation in the electrolyte, which exacerbates their shuttling effect, thereby causing severe capacity degradation.^[^ ^2^ ^]^ An electrocatalytic approach to accelerating LiPSs redox kinetics seems to be a natural strategy for suppressing the shuttling effect and enhancing electrochemical cycling stability.^[^ ^3^ ^]^ Transition metal compounds such as metal oxides,^[^ ^4^ ^]^ sulfides,^[^ ^5^ ^]^ phosphides,^[^ ^6^ ^]^ and their heterostructures^[^ ^7^ ^]^ have been introduced to accelerate the sulfur reduction reaction (SRR) and alleviate LiPSs shuttle. Among these, Mn‐based catalysts have attracted attention because of their strong interactions with LiPSs, and they have been widely used to inhibit soluble LiPSs shuttling.^[^ ^8^ ^]^ Nazar et al. reported that δ‐MnO_2_ nanosheets first oxidized LiPSs to form thiosulfates, and the generated thiosulfates quickly converted LiPSs to polythionate complexes and Li_2_S.^[^ ^9^ ^]^ Shang et al. revealed that Mn_2_P, owing to its polar nature, exhibited strong chemical adsorption of LiPSs and enhanced Li_2_S nucleation growth kinetics.^[^ ^10^ ^]^ Zhang et al. fabricated a Mn_3_O_4_‐MnP_x_ heterostructure to combat the shuttling effect of LiPSs, in which Mn_3_O_4_ strongly anchors the LiPSs and MnP_x_ can significantly catalyze LiPSs conversion; the synergistic effect of Mn_3_O_4_ and MnP_x_ ensured the trapping‐catalytic conversion of LiPSs and accelerated the redox kinetics.^[^ ^11^ ^]^ Although no MnO_2_‐manganese phosphides have been reported for SRR, these findings encouraged us to believe that the MnP‐MnO_2_ heterostructure can effectively inhibit LiPSs shuttle.

Notably, the surface of the electrocatalysts is likely to be etched to produce sulfidation in the LiPSs‐rich aprotic environment, leading inevitably to different electrocatalytic behaviors.^[^ ^12^ ^]^ Capturing and understanding the catalyst sulfidation is crucial for understanding the SRR catalytic process. Recently, Huang et al. demonstrated that the low‐valence Co metal atom in Co_4_N was initially etched by LiPSs, which promoted Co vacancy formation and facilitated the formation of CoS_x_ phases.^[^ ^13^ ^]^ Our recent work also observed sulfidation of the VC‐VO heterostructure during sulfur redox reactions.^[^ ^14^ ^]^ The V_5_S_8_ phase formed by sulfidation exhibited excellent catalytic activity and accelerated LiPSs conversion kinetics. However, the Mn‐based catalyst sulfidation mechanism remains unexplored and requires further investigation.

**Figure 1:**
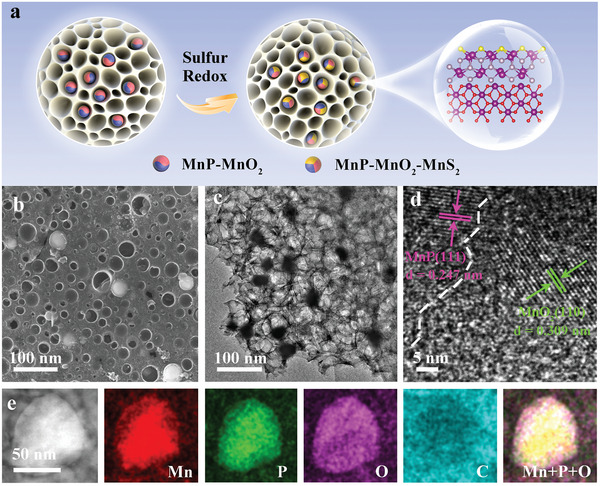
a) Surface sulfidation of the MnP‐MnO_2_ heterostructure during the sulfur redox process. b) SEM, c) TEM, and d) HRTEM images of MnP‐MnO_2_/C. e) HAADF–STEM and EDS element distribution mapping for MnP‐MnO_2_/C.

In this study, we synthesized heterogeneous MnP‐MnO_2_ nanoparticles anchored in porous carbon (MnP‐MnO_2_/C) via a one‐step heat treatment of Mn^2+^‐exchange resins with KOH. More importantly, a novel MnS_2_ phase on the MnP‐MnO_2_ surface was observed during the sulfur redox process (**Figure** **1**a) using characterization methods such as in situ XRD, in situ Raman spectroscopy, and TEM before and after charging/discharging. Furthermore, DFT calculations showed that MnS_2_ tended to form on the MnP surface layer of the MnP‐MnO_2_ heterostructure. Surface sulfidation endows the MnP‐MnO_2_ heterostructure electrocatalyst with excellent electrocatalytic activity, greatly accelerating the SRR kinetics and promoting Li_2_S reoxidation. Benefiting from this, the Li─S batteries equipped with the MnP‐MnO_2_/C@S cathode exhibited remarkable cycling stability and rate performance. Moreover, working Li−S pouch cells with an actual energy density of 429 Wh kg^−1^ demonstrate the practical potential of surface sulfidation.

## 2 Results and Discussion

### 2.1 Material Synthesis and Characterization

MnP‐MnO_2_/C, MnP/C, and MnO_2_/C were synthesized by one‐step heat treatment of the mixture of Mn^2+^‐containing resin and potassium hydroxide. The X‐ray diffraction (XRD) showed that MnP‐MnO_2_/C contained two components of MnP and MnO_2_ (Figure S1, Supporting Information). The characteristic peaks located at 32.0°, 36.4°, 45.3°, 46.2°, and 47.5° were indexed to the (101), (111), (211), (220), and (121) planes of MnP (JCPDS NO. 51–0942), respectively. The diffraction peaks at 28.8°, 37.7°, 40.6°, and 59.8° are ascribed to the (110), (011), (200), and (220) planes of MnO_2_ (JCPDS NO. 50–0866), respectively. The SEM image of MnP‐MnO_2_/C shows a porous framework structure and heterogeneous MnP‐MnO_2_ nanoparticles with an average size of ≈50 nm. These nanoparticles were homogeneously embedded in the porous carbon (Figure 1b). The TEM image of MnP‐MnO_2_/C further illustrates that numerous nanoparticles are uniformly distributed in the hierarchical porous carbon network (Figure 1c). High‐resolution TEM (HRTEM) image confirms that the lattice fringes at 0.247 and 0.309 nm correspond to the MnP (111) and MnO_2_ (110) planes, respectively (Figure 1d). There is an obvious heterointerface between MnP and MnO_2_ that may promote electron transport and strengthen LiPSs adsorbability by increasing the number of reactive sites, similar to other heterogeneous interfaces.^[^ ^3^, ^15^ ^]^ EDS showed the coexistence of Mn, P, and O within the heterogeneous MnP‐MnO_2_ nanoparticles (Figure 1e). Notably, the C element was located around the nanoparticles.

These results consistently indicate that heterogeneous MnP‐MnO_2_ nanoparticles were successfully synthesized and uniformly anchored in porous carbon. The as‐prepared MnP‐MnO_2_/C was conducive to improving electron transfer and electrolyte wetting. The structures of MnP/C, MnO_2_/C, and pure C are in good agreement with those of MnP‐MnO_2_/C (Figure S2, Supporting Information). The N_2_ adsorption–desorption isotherms and pore‐size distribution curves of MnP‐MnO_2_/C, MnP/C, and MnO_2_/C exhibit distinct micro/mesoporous structures, with Brunauer–Emmett–Teller (BET) surface areas of 280.4, 223.6, and 203.1 m^2^ g^−1^ for MnP‐MnO_2_/C, MnP/C, and MnO_2_/C, respectively (Figure S3, Supporting Information). This porous structure and large specific surface area favor the storage of sulfur species.^[^ ^16^ ^]^

### 2.2 The Adsorption Process toward LiPSs

**Figure 2:**
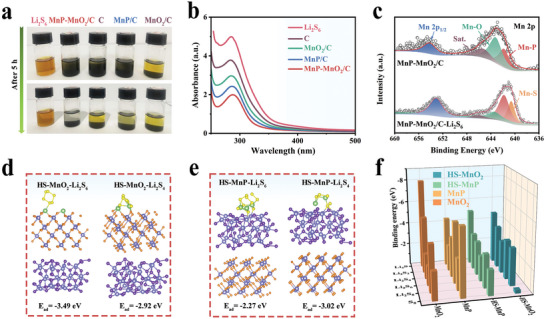
a) Photographs of the adsorption measurement and b) corresponding UV‐Vis spectra of Li_2_S_6_ after 5 h adsorption by MnP‐MnO_2_/C, MnP/C, MnO_2_/C, and pure C. c) XPS spectra of Mn 2p for MnP‐MnO_2_/C before and after Li_2_S_6_ adsorption. Optimized adsorption configurations of Li_2_S_6_ and Li_2_S_4_ on d) the (110) plane of MnO_2_ in MnP‐MnO_2_ and e) the (111) plane of MnP in MnP‐MnO_2_, respectively. f) Calculated binding energies of S_8_ and lithium polysulfide species (Li_2_S, Li_2_S_2_, Li_2_S_4_, Li_2_S_6_, and Li_2_S_8_) adsorbed on the surfaces of MnO_2_, MnP, and MnP‐MnO_2_, respectively.

As shown in **Figure** **2**a, Li_2_S_6_ solution was used as a representative soluble LiPSs to evaluate the adsorption capacity of MnP‐MnO_2_/C. After 5 h, the solution immersed in MnP‐MnO_2_/C became visibly transparent, whereas the solutions containing MnP/C and MnO_2_/C remained yellow. Subsequently, the supernatant was characterized using UV–vis spectroscopy. The strong absorption peak at 250–350 nm indicates the presence of Li_2_S_6_ (Figure 2b).^[^ ^17^ ^]^ Notably, the solution containing MnP‐MnO_2_/C exhibited a significantly lower absorbance in this region than solutions containing MnP/C, MnO_2_/C, and pure C. These results indicate the much higher adsorbability of MnP‐MnO_2_/C toward LiPSs capture. The survey X‐ray photoelectron spectroscopy (XPS) spectra illustrate the surface chemical states of MnP‐MnO_2_/C after the Li_2_S_6_ adsorption measurement (Figure 2c), which is labeled as MnP‐MnO_2_/C−Li_2_S_6_. Deconvolution of the Mn 2p_3/2_ spectrum of MnP‐MnO_2_/C revealed that it was primarily composed of Mn─O (643.3 eV) and Mn─P (641.8 eV).^[^ ^18^ ^]^ In addition, a prominent peak was observed at a higher binding energy (645.5 eV), corresponding to a satellite peak. After adsorption, an additional Mn 2p_3/2_ peak appeared at a lower energy (640.6 eV), which can be attributed to Mn‐S.^[^ ^19^ ^]^ The S 2p spectrum reveals four sulfur environments (Figure S4, Supporting Information); those at 162.8 and 164.1 eV belong to “bridging” (S_B_^0^) and “terminal” (S_T_^−1^) sulfur.^[^ ^20^ ^]^ Two sulfur environments at 168–170 eV were observed and attributed to the thiosulfate and polythionate complexes, consistent with a previous report.^[^ ^21^ ^]^ The formation of thiosulfate/polythionate can be attributed to MnO_2_ oxidizing LiPSs to form thiosulfate groups, which further convert thiosulfate to polythionate complexes and short‐chain Li_2_S. The significant variations in the XPS spectra of MnP‐MnO_2_/C before and after the Li_2_S_6_ adsorption test clearly demonstrate the chemical interaction between MnP‐MnO_2_/C and Li_2_S_6_. DFT calculations were used to assess the binding energies of the LiPSs on the surfaces of MnP (111)‐MnO_2_ (110), MnP (111), and MnO_2_ (110). The calculated adsorption configurations of MnP‐MnO_2_ are illustrated in Figure 2d,e, and Figure S5 (Supporting Information). The results suggested that the S and Li atoms in Li_2_S_6_ and Li_2_S_4_ preferentially bind with Mn on the MnP side and O atoms on the MnO_2_ side of the MnP (111)‐MnO_2_ (110) heterostructure. As shown in Figure 2f, compared with MnP and MnO_2_, MnP‐MnO_2_ showed moderate binding energy for LiPSs adsorption, which is advantageous for promoting the adsorption of LiPSs and the subsequent catalytic sulfur reduction reaction.^[^ ^22^ ^]^

### 2.3 Electrocatalytic Behavior of the Catalysts

**Figure 3:**
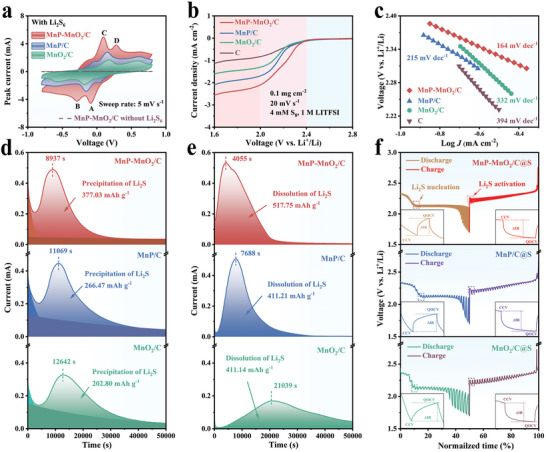
a) CV profiles of symmetrical cells at a scan rate of 5 mV s^−1^ with MnP‐MnO_2_/C, MnP/C, MnO_2_/C, and MnP‐MnO_2_/C without Li_2_S_6_. b) LSV curves and c) corresponding Tafel plots of MnP‐MnO_2_/C, MnP/C, and MnO_2_/C. d) Potentiostatic discharge profiles of Li_2_S nucleation at 2.05 V and e) potentiostatic charge profiles of Li_2_S activation at 2.35 V for MnP‐MnO_2_/C, MnP/C, and MnO_2_/C. f) GITT curves of MnP‐MnO_2_/C@S, MnP/C@S, and MnO_2_/C@S.

To estimate the electrocatalytic behavior of different electrocatalysts in the LiPSs redox reaction process, cyclic voltammetry (CV) profiles of the symmetrical cell based on Li_2_S_6_ solution at a scan rate of 5 mV s^−1^ were tested (**Figure** **3**a). The curve of MnP‐MnO_2_/C clearly displays two pairs of reversible redox peaks, labeled A, B, C, and D. The CV curve of MnP‐MnO_2_/C without Li_2_S_6_ solution exhibited capacitive behavior.^[^ ^23^ ^]^ In general, peak A represents the reduction of Li_2_S_6_ to Li_2_S_4_, peak B suggests the conversion of Li_2_S_4_ to Li_2_S_2_/Li_2_S, and peaks C and D represent the reverse processes of peaks A and B, respectively.^[^ ^24^ ^]^ MnP‐MnO_2_/C exhibited significantly enhanced current densities for all redox peaks compared to MnP/C and MnO_2_/C, suggesting that LiPSs conversion using MnP‐MnO_2_/C had much faster reaction kinetics. Importantly, the quantitative results also show that the MnP‐MnO_2_/C electrocatalyst displays a much smaller polarization voltage (0.09 mV) than MnP/C (0.16 mV) and MnO_2_/C (0.18 mV), indicating a lower energy barrier toward Li_2_S_6_ conversion (Figure S6, Supporting Information).

A three‐electrode system was developed to examine the electrocatalytic performance of the different electrocatalysts in the SRR. As illustrated in the linear sweep voltammetry (LSV) curves (Figure 3b), a deformed “S‐shaped” region can be observed and divided into kinetic‐controlled, mixed‐controlled, and diffusion‐controlled regions.^[^ ^25^ ^]^ In the high‐potential area, the limiting factor for the overall electrocatalytic performance is the interfacial reaction kinetics; in such a potential range, the overpotential is insufficient to overcome the reaction barrier and effect SRR. In the low‐potential region, the larger overpotential makes LiPSs conversion fast enough so that mass diffusion becomes the limiting factor, and the current changes slightly with increased overpotential; this is called the diffusion‐controlled region. In the intermediate‐potential region, known as the mixed‐controlled region, the SRR is co‐dominated by surface reactions and mass diffusion. LSV curves are similar to those of the oxygen reduction reaction, including the half‐wave potential (*E_1/2_*), onset potential, and diffusion‐limited current density (*J_d_*).^[^ ^26^ ^]^ Surprisingly, MnP‐MnO_2_/C exhibited the highest onset potential, *E_1/2_*, and *J_d_*, indicating that the MnP‐MnO_2_/C electrode had superior electrocatalytic activity compared with the MnP/C, MnO_2_/C, and pure C electrodes. Furthermore, the Tafel slope (*η*) and the exchange current density (*J_0_*) determined from the LSV curve provide the key kinetic parameters that characterize the reaction kinetics and electrocatalytic activity of the electrocatalysts.^[^ ^27^ ^]^ As shown in Figure 3c, the MnP‐MnO_2_/C electrocatalyst exhibits the smallest *η* of 164 mV dec^−1^ compared to 215, 332, and 384 mV dec^−1^ for MnP/C, MnO_2_/C, and pure C, respectively, indicating accelerated reaction kinetics and higher electrocatalytic activity. By extrapolating the Tafel plot to zero overpotential, a *J_0_* value of 0.104 mA cm^−2^ is obtained for the MnP‐MnO_2_/C electrocatalyst, which is higher than those of MnP/C (0.094 mA cm^−2^), MnO_2_/C (0.093 mA cm^−2^), and pure C (0.086 mA cm^−2^). The smaller *η* and the higher *J_0_* are important indicators of faster reaction kinetics.

To evaluate the deposition and dissolution kinetics of the Li_2_S conversion precisely, potentiostatic discharge/charge measurements at 2.05/2.35 V were performed. As illustrated in Figure 3d, the MnP‐MnO_2_/C electrocatalyst demonstrated a higher peak current and reached the current peak at 8937 s, which was considerably faster than those of MnP/C (11 069 s) and MnO_2_/C (12 642 s). Meanwhile, the calculated capacity contribution of Li_2_S deposition is 377.03 mAh g^−1^ for MnP‐MnO_2_/C, while the capacity values of MnP/C and MnO_2_/C are 266.47 and 202.80 mAh g^−1^, respectively. In the case of Li_2_S dissolution (Figure 3e), the MnP‐MnO_2_/C electrocatalyst reached its current peak in a shorter time (4055 s) than MnP/C (7688 s) and MnO_2_/C (21 039 s). More impressively, the Li_2_S dissolution capacity on MnP‐MnO_2_/C is 517.75 mAh g^−1^, which outperforms the capacities on MnP/C (411.21 mAh g^−1^) and MnO_2_/C (411.14 mAh g^−1^). Notably, MnO_2_/C exhibits a capacity for Li_2_S dissolution comparable to that of MnP/C. However, the time required to reach the peak was 2.4 times longer for MnO_2_/C than for MnP/C. This discrepancy may be attributed to the strong adsorption of Li_2_S by MnO_2_, which hindered the oxidation of Li_2_S. These results indicate that MnP‐MnO_2_/C can accelerate SRR kinetics and promote Li_2_S activation.

To further evaluate the electrocatalytic ability of different electrocatalysts in promoting Li_2_S nucleation and activation, galvanostatic intermittent titration technique (GITT) profiles were measured at 0.05 C using Li−S coin cells equipped with MnP‐MnO_2_/C@S, MnP/C@S, and MnO_2_/C@S cathodes. The dip depth of the discharge/charge curves indicated the cathode internal resistances to the nucleation and activation of Li_2_S (Figure 3f). The internal resistance *ΔR_internal_* value of the MnP‐MnO_2_/C@S cathode for Li_2_S nucleation and activation is smaller than those of the MnP/C@S and MnO_2_/C@S cathodes, indicating that the MnP‐MnO_2_/C@S cathode promoted redox kinetics and increased LiPSs conversion ability.^[^ ^28^ ^]^

### 2.4 Exploration of the Surface Sulfidation

**Figure 4:**
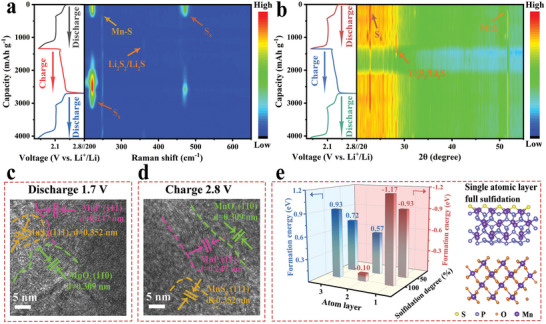
a) In situ Raman spectra and b) in situ XRD spectra of the MnP‐MnO_2_/C@S cathode at 0.2 C and corresponding discharge/charge curves. HRTEM images of MnP‐MnO_2_/C@S cathodes after c) discharging to 1.7 V, and d) charging to 2.8 V. e) The formation energy of MnS_2_ after the interaction between MnP‐MnO_2_ and LiPSs and corresponding optimized configurations of single atomic layer full sulfidation.

To further investigate the SRR details of the MnP‐MnO_2_/C@S cathode inside Li─S batteries in real time, we performed in situ Raman spectroscopy (**Figure** **4**a).^[^ ^29^ ^]^ In situ observation showed that the two characteristic signals at 221 and 471 cm^−1^ belong to S_8_.^[^ ^30^ ^]^ During the discharge process, S_8_ was converted into soluble long‐chain LiPSs, and its characteristic signal intensity gradually weakened. At 2.1 V, the S_8_ signal disappeared, indicating complete conversion. At a voltage of 2.0–1.7 V, a new characteristic signal appeared near 360 cm^−1^, corresponding to the generation of insoluble Li_2_S_2_/Li_2_S. Notably, at a discharge voltage of 2.2 V, an unprecedented characteristic signal appeared (248 cm^−1^), representing the Mn─S signal.^[^ ^31^ ^]^ During charging, the Li_2_S_2_/Li_2_S signal began to disappear, and the S_8_ signal reappeared, implying that the insoluble short‐chain Li_2_S_2_/Li_2_S was gradually converted to long‐chain LiPSs and finally to solid S_8_. The periodic evolution of sulfur species corresponded to a typical solid‐liquid‐solid conversion. In situ XRD analysis further confirmed the occurrence of sulfidation in the MnP‐MnO_2_/C@S cathode (Figure 4b). During the charging and discharging processes, the S_8_ signal disappeared and reappeared regularly, whereas the Li_2_S signal (PDF#23‐0369) was generated and decreased regularly, corresponding to the reversible transformation of the sulfur species.^[^ ^32^ ^]^ Interestingly, at a discharge voltage of 2.2 V, a new characteristic signal appeared at 51.8°, which was attributed to MnS_2_ (PDF#25‐0549).^[^ ^33^ ^]^ This is consistent with the in situ Raman results.

To further confirm the presence of the MnS_2_ phase and its stability during charging and discharging, we used HRTEM to characterize the MnP‐MnO_2_/C@S cathode discharged at 1.7 V and charged at 2.8 V. As shown in Figure 4c, when discharged to 1.7 V, the HRTEM image showed the coexistence of three‐phase lattice fringes corresponding to the MnP (111), MnO_2_ (110), and MnS_2_ (111) lattice planes. Similarly, when charged to 2.8 V, the MnP, MnO_2_, and MnS_2_ phases were clearly observed (Figure 4d). The HRTEM results indicated that the MnP‐MnO_2_ catalyst underwent partial sulfidation, forming a novel MnS_2_ phase. Moreover, the MnS_2_ phase retained good stability during the electrochemical process. To obtain a comprehensive understanding of the sulfidation mechanism, a DFT analysis was carried out. As shown in Figure 4e and Figure S7 (Supporting Information), sulfidation is prone to occur on the MnP side of MnP‐MnO_2_, containing low‐valence Mn atoms, because LiPSs easily etch low‐valence metal atoms to form M‐S species, which then evolve into the corresponding sulfides.^[^ ^13^, ^19^ ^]^ Moreover, the sulfidation of the single atomic layer in MnP‐MnO_2_ is thermodynamically easier to form and more stable, especially after all the P atoms in the surface layer are sulfurized and the formation energy (−1.17 eV) of the system is lowest. More importantly, the MnS_2_ phase maintains high stability during electrochemical reactions, and according to previous reports, sulfidation of the electrocatalyst is expected to improve the electrocatalytic activity and promote rapid redox kinetics.^[^ ^34^ ^]^

### 2.5 Improvement of Electrocatalysis after Surface Sulfidation

**Figure 5:**
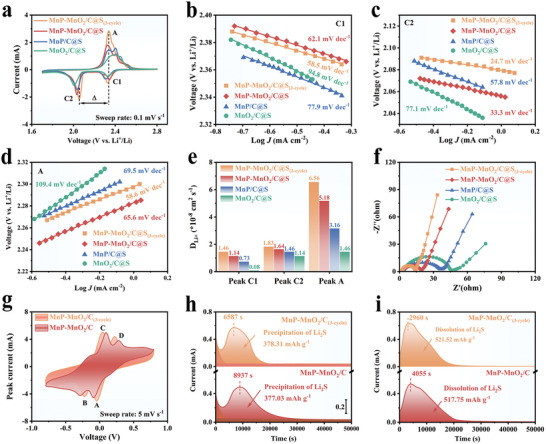
a) CV profiles at a sweep rate of 0.1 mV s^−1^ for MnP‐MnO_2_/C@S_(3‐cycle)_, MnP‐MnO_2_/C@S, MnP/C@S, and MnO_2_/C@S cathodes. b−d) Tafel plots and e) Li^+^ diffusion coefficients of peaks C1, C2, and A. f) Nyquist curves of MnP‐MnO_2_/C@S_(3‐cycle)_, MnP‐MnO_2_/C@S, MnP/C@S, and MnO_2_/C@S cathodes. g) CV profiles of Li_2_S_6_ symmetrical cells for MnP‐MnO_2_/C_(3‐cycle)_ and MnP‐MnO_2_/C electrodes at a scan rate of 5 mV s^−1^. h) Potentiostatic discharge profiles of Li_2_S nucleation at 2.05 V and i) potentiostatic charge profiles of Li_2_S activation at 2.35 V for MnP‐MnO_2_/C@S_(3‐cycle)_ and MnP‐MnO_2_/C@S cathodes.

All the sulfur cathode CV curves clearly display two cathodic peaks (C1 and C2) and an overlapping anodic peak (A), as shown in **Figure** **5**a. Compared to the MnP/C@S and MnO_2_/C@S cathodes, the C1 and C2 peaks of the MnP‐MnO_2_/C@S cathode were shifted to higher potentials, suggesting a rapid transformation from S_8_ molecules to soluble LiPSs and accelerated reduction kinetics from LiPSs to insoluble Li_2_S. Simultaneously, peak A shifted to a lower potential, implying that the oxidation process was promoted as Li_2_S converted to LiPSs and ultimately to S_8_. Moreover, peaks C1, C2, and A of the MnP‐MnO_2_/C@S cathode exhibit a higher current response than those of the MnP/C@S and MnO_2_/C@S cathodes, indicating efficient SRR and Li_2_S oxidation. Interestingly, among all the cathodes, the MnP‐MnO_2_/C@S_(3‐cycle)_ cathode exerts the lowest potential difference (*Δ* = 282 mV) and highest redox peak intensities (Table S1, Supporting Information), which can be attributed to the boosted redox kinetics and improved sulfur species conversion efficiency resulting from the surface sulfidation. In addition, the Tafel slopes of the C1, C2, and A peaks were derived to quantify the electrocatalytic activity. As depicted in Figure 5b−d, the Tafel slopes of the MnP‐MnO_2_/C@S_(3‐cycle)_ cathode reached 58.5, 24.7, and 58.6 mV dec^−1^, respectively. These slopes were lower than those of MnP‐MnO_2_/C@S, MnP/C@S, and MnO_2_/C@S cathodes, again implying the MnP‐MnO_2_/C@S_(3‐cycle)_ cathode improved electrocatalytic behavior. Moreover, the MnP‐MnO_2_/C@S_(3‐cycle)_ cathode CV curves remained unchanged after four cycles, indicating its remarkable electrochemical cycle stability (Figure S8, Supporting Information). In contrast, the peak trends of the MnP‐MnO_2_/C@S cathode after four cycles fluctuated slightly, especially after the first cycle, when the redox peak current responses increased slightly and the polarization decreased slightly. This result may be due to the surface sulfidation, which enhances the electrocatalytic activity of the MnP‐MnO_2_/C@S cathode.

The lithium‐ion (Li^+^) diffusion rate, another vital factor affecting LiPSs conversion kinetics, was investigated by analyzing the CV curves. As shown in Figure 5e, the calculated Li^+^ diffusion coefficients of peaks C1, C2, and A for the MnP‐MnO_2_/C@S_(3‐cycle)_ cathode are 1.46 × 10^−8^, 1.83 × 10^−8^, and 6.56 × 10^−8^ cm^2^ s^−1^, respectively, which are significantly higher than the values obtained for MnP‐MnO_2_/C@S, MnP/C@S, and MnO_2_/C@S cathodes. These high diffusivities indicate that surface sulfidation endows the MnP‐MnO_2_/C electrocatalyst with significant electrocatalytic activity and improved LiPSs conversion kinetics. Electrochemical impedance spectroscopy (EIS) was also used to qualitatively investigate the charge transfer resistance (*R_ct_*) and diffusion ability of Li^+^. As shown in Figure 5f, all the Nyquist plots show a semicircle and a sloping line, where the semicircle in the high‐frequency region corresponds to *R_ct,_* and the linear feature in the low‐frequency region is associated with Li^+^ diffusion.^[^ ^35^ ^]^ The *R_ct_* of the MnP‐MnO_2_/C@S_(3‐cycle)_ cathode is 12.20 Ω, which is much lower than those of the MnP‐MnO_2_/C@S, MnP/C@S, and MnO_2_/C@S cathodes (Table S2, Supporting Information). The steepest slope of the MnP‐MnO_2_/C@S_(3‐cycle)_ cathode indicated the best Li^+^ diffusion. These results further confirmed that the MnP‐MnO_2_ heterostructure electrocatalyst, after surface sulfidation, could accelerate Li^+^ transport and promote the catalytic conversion of LiPSs.^[^ ^36^ ^]^

In addition, the symmetrical cell of the MnP‐MnO_2_/C@S_(3‐cycle)_ cathode exhibited a peak shape similar to that of the MnP‐MnO_2_/C@S cathode (Figure 5g). However, the MnP‐MnO_2_/C@S_(3‐cycle)_ cathode exhibited a higher current peak intensity and a shorter time to reach the peak, indicating improved electrocatalytic activity and enhanced redox reaction kinetics toward LiPSs conversion. Furthermore, the Li_2_S deposition results showed that the time required for the MnP‐MnO_2_/C@S_(3‐cycle)_ cathode to reach the peak current was 6587 s, which was 1.36 times shorter than that of the MnP‐MnO_2_/C@S cathode (Figure 5h). The MnP‐MnO_2_/C@S_(3‐cycle)_ cathode also exhibited a higher peak current intensity and a larger deposition capacity (378.31 mAh g^−1^) than did the MnP‐MnO_2_/C@S cathode. The Li_2_S dissolution measurement reveals that the MnP‐MnO_2_/C@S_(3‐cycle)_ cathode exhibits a higher current response, a faster speed to reach the peak current (2960 s), and a larger deposition capacity (521.52 mAh g^−1^) than that of the MnP‐MnO_2_/C@S cathode (Figure 5i), suggesting faster and more effective Li_2_S activation. These results demonstrate that surface sulfidation significantly improves the MnP‐MnO_2_ heterostructure electrocatalyst promotion of LiPSs catalytic conversion and Li_2_S activation.

### 2.6 Electrochemical Performance of Li─S Batteries

**Figure 6:**
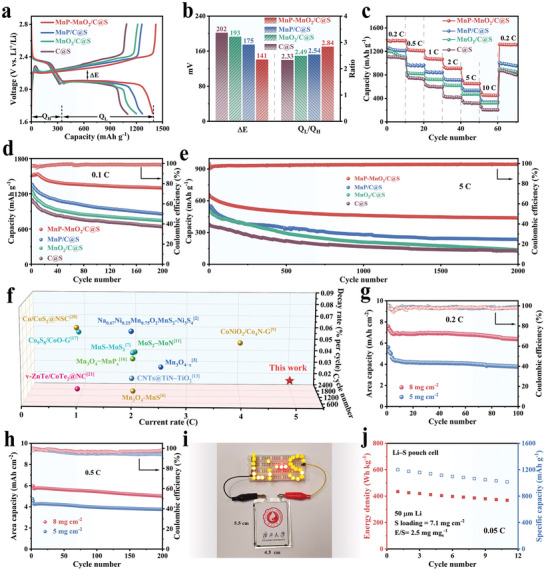
a) GCD profiles at 0.2 C, b) *ΔE* and *Q_L_/Q_H_* ratio obtained from GCD curves, c) rate performance at different current densities, d) cycling performance at 0.2 C, and e) long‐term cycling performance at 5 C of MnP‐MnO_2_/C@S, MnP/C@S, MnO_2_/C@S, and C@S cathodes. f) Performance comparison of the MnP‐MnO_2_/C@S cathode with recent reported works. Cycling performance of MnP‐MnO_2_/C@S with sulfur loading of 5 and 8 mg cm^−2^ at g) 0.2 C and h) 0.5 C. i) Photograph of a panel with an “Li−S” pattern illuminated by the pouch cell with MnP‐MnO_2_/C@S cathode. j) Cycling performance of Li−S pouch cell equipped with MnP‐MnO_2_/C@S cathode.

To evaluate the improvement of the MnP‐MnO_2_/C@S cathode (sulfur content 78.2 wt%, Figure S9, Supporting Information) on the electrochemical performance of Li─S batteries, coin cells with a sulfur loading of 1.8 mg cm^−2^ were assembled. The galvanostatic charge–discharge (GCD) curves of all cathodes at 0.2 C exhibited two separate discharge plateaus and a continuous charge plateau, consistent with the CV results (**Figure** **6**a). The voltage gap between the charge plateau and the second discharge plateau is generally referred to as the polarization potential (*ΔE*).^[^ ^37^ ^]^ As displayed in Figure 6a,b, the MnP‐MnO_2_/C@S cathode showed a lower *ΔE* of 141 mV compared to the MnP/C@S, MnO_2_/C@S, and C@S cathodes. The solid–liquid–solid conversion of sulfur species on the MnP‐MnO_2_/C@S cathode was further measured using the *Q_L_/Q_H_* ratio; a higher *Q_L_/Q_H_* ratio indicates better catalytic ability because the shuttling effect of soluble LiPSs leads to capacity fading during the *Q_L_* stage.^[^ ^38^ ^]^ The MnP‐MnO_2_/C@S cathode exhibited a *Q_L_/Q_H_* value of 2.84 (Figure 6b), which was larger than those of the MnP/C@S, MnO_2_/C@S, and C@S cathodes. The ideal *Q_L_/Q_H_* ratio indicating complete conversion of S_8_ to Li_2_S is 3.0, making a ratio of 2.84 equivalent to a 94.7% conversion fraction, a result that suggests ultrafast SRR kinetics. In addition, the MnP‐MnO_2_/C@S cathode delivered the lowest overpotential (16.5 mV) during the initial charging stage (Figure S10, Supporting Information), representing a lower activation energy barrier and promoting Li_2_S decomposition, consistent with the experimental results of Li_2_S dissolution. Furthermore, the MnP‐MnO_2_/C@S cathode exhibited impressive rate capacities of 1380 mAh g^−1^ at 0.2 C and 500 mAh g^−1^ even at 10 C, respectively, significantly better than those of the MnP/C@S, MnO_2_/C@S, and C@S cathodes (Figure 6c). More importantly, when the current density was switched back to 0.2 C, the MnP‐MnO_2_/C@S cathode was still able to output a high discharge capacity of 1321 mAh g^−1^ with a capacity retention of 95.7%. Such excellent rate performance implies that the MnP‐MnO_2_/C@S cathode still exhibits fast electron transfer and accelerated LiPSs conversion at high current rates. Additionally, the cycling performance of the different cathodes at 0.1 C is illustrated in Figure 6d. The MnP‐MnO_2_/C@S cathode delivered an initial discharge capacity of 1511 mAh g^−1^ and maintained a capacity retention rate of 86% after 200 cycles. Notably, the discharge capacity of the MnP‐MnO_2_/C@S cathode remained essentially unchanged during the first few cycles, which may be attributed to the MnS_2_ phase formed by surface sulfurization, which enhanced the SRR kinetics. In contrast, the MnP/C@S, MnO_2_/C@S, and C@S cathodes exhibited lower initial discharge capacities of 1360, 1247, and 1097 mAh g^−1^, with capacity retention of 62.8%, 59.5%, and 58.3%, respectively. The MnP‐MnO_2_/C@S cathode exhibited an admirable initial discharge capacity of 815 mAh g^−1^ with a tiny capacity decay rate of 0.021% per cycle after 1200 cycles at 2 C (Figure S11, Supporting Information), compared to MnP/C@S (766.6 mAh g^−1^, 0.030%), MnO_2_/C@S (706.6 mAh g^−1^, 0.043%), and C@S (502.5 mAh g^−1^, 0.062%) cathodes. Furthermore, long‐term cycling performance was achieved at 5 C (Figure 6e). The MnP‐MnO_2_/C@S cathode displayed an initial discharge capacity of 656.5 mAh g^−1^, with a capacity attenuation of 0.017% per cycle after 2000 cycles. In contrast, the capacity decay rates of the MnP/C@S, MnO_2_/C@S, and C@S cathodes were 0.028%, 0.033%, and 0.037%, respectively. The electrochemical performance of the MnP‐MnO_2_/C@S cathodes at 5 C was superior to that of most reported Li─S batteries (Figure 6f; Table S3, Supporting Information). The outstanding electrochemical performance of the MnP‐MnO_2_/C@S cathode can be attributed to the significantly improved SRR kinetics of the MnP‐MnO_2_ heterostructure after surface sulfidation, which promotes the rapid conversion of LiPSs and effectively inhibits their shuttling effect.

The superiority of the MnP‐MnO_2_/C electrocatalyst is further validated under more practical conditions; the performances of MnP‐MnO_2_/C@S cathodes with high sulfur loadings of 5.0 and 8.0 mg cm^−2^ were evaluated. As shown in Figure 6g, at a current density of 0.2 C, the MnP‐MnO_2_/C@S cathode provided an initial areal capacity of 5.6 mAh cm^−2^ with a high sulfur loading of 5 mg cm^−2^. After 100 cycles, the cathode still maintained a high areal capacity of 3.8 mAh cm^−2^. More importantly, even when the sulfur loading of the MnP‐MnO_2_/C@S cathode was increased to 8.0 mg cm^−2^, the cell still achieved a high initial capacity of 7.6 mAh cm^−2^ and maintained a high capacity retention rate of 84.2% after 100 cycles. When cycling tests were conducted at a higher current intensity of 0.5 C (Figure 6h), the MnP‐MnO_2_/C@S cathode, with areal sulfur loadings of 5.0 and 8.0 mg cm^−2^, exhibited attractive initial areal capacities of 4.8 and 6.0 mAh cm^−2^ and achieved a tiny capacity attenuation rate of 0.110% and 0.083% after 200 cycles, respectively. These results show that even under high sulfur loading, the MnP‐MnO_2_/C@S cathode exhibits outstanding electrochemical performance, which can be ascribed to the enhanced redox kinetics for LiPSs conversion and improved ability to capture LiPSs.

To validate the utility of the MnP‐MnO_2_/C@S cathode in practical devices, a Li─S pouch cell was assembled. Ultrathin Li metal anodes (50 µm), low E/S ratio (5.0 µL mg^−1^), and high‐loading sulfur cathodes (7.1 mg cm^−2^) were employed. This Li−S pouch cell was able to illuminate a panel with a “Li−S” pattern (Figure 6i). Furthermore, the Li─S pouch cell exhibited a high initial discharge capacity of 1199 mAh g^−1^ at 0.05 C and displayed stable performance for 11 cycles, with a capacity retention of 84.7% (Figure 6j; Figure S12, Supporting Information). More importantly, it also delivered an initial energy density of 429 Wh kg^−1^ and still maintained a high energy density of 362.3 Wh kg^−1^ after cycling. The effectiveness of the MnP‐MnO_2_ heterostructure electrocatalyst was successfully validated by the excellent electrochemical performance advantages in both coin and pouch cells.

## 3 Conclusion

We have developed a MnP‐MnO_2_ heterostructure catalyst in Li─S batteries for accelerated sulfur reduction reaction (SRR) kinetics and inhibited shuttling effect by using a one‐step heat treatment method. Excitingly, a new MnS_2_ phase was generated on the MnP‐MnO_2_ surface during charge and discharge, which was confirmed by density functional theory (DFT) calculations, in situ XRD and in situ Raman spectroscopy, and TEM before and after charge and discharge. More importantly, surface sulfidation of the MnP‐MnO_2_ heterostructure catalyst significantly accelerated the SRR and Li_2_S activation, thereby improving the utilization of the active material. The Li−S batteries equipped with MnP‐MnO_2_/C@S cathode exhibited excellent rate performance (10 C, 500 mAh g^−1^) and ultrahigh cycling stability (0.017% decay rate per cycle at 5 C). A pouch cell with MnP‐MnO_2_/C@S cathode delivers a high energy density of 429 Wh kg^−1^, showing good potential for commercial applications.

## Conflict of Interest

The authors declare no conflict of interest.

## Supporting information

Supporting Information

## Data Availability

The data that support the findings of this study are available from the corresponding author upon reasonable request.
