# Supplementary material for: Mastering Surface Sulfidation of MnP‐MnO2 Heterostructure to Facilitate Efficient Polysulfide Conversion in Li─S Batteries
Source: Adv Sci (Weinh). 2024 Jun 24;11(32):2403391. doi: 10.1002/advs.202403391 (PMC11348264; doi:10.1002/advs.202403391)
Supplement: Supplementary file 1 — Supporting Information [file ADVS-11-2403391-s001.docx]

Supporting Information

Mastering Surface Sulfidation of MnP-MnO_2_ Heterostructure to Facilitate Efficient Polysulfide Conversion in Li–S Batteries

*Fengxing Liang, Qiao Deng,* *Shunyan Ning*,* *Huibing He, Nannan Wang, Yanqiu Zhu*, Jinliang Zhu**

# Experimental Section

**Synthesis of Materials**

*Preparation of Precursor:* 50 g of the phosphorus-containing resin was added into 250 mL of deionizsed water and stirred uniformly, and manganese chloride tetrahydrate (Mn(CH_3_COO)_2_) (Shanghai Aladdin Biochemical Technology Co. Ltd., China) was dissolved into deionizsed water to formulate a Mn^2+^ solution of 0.05 mmol, which was then added into the phosphorus-containing resin, and stirred uniformly at 60 ℃ for 6 h. The Mn^2+^-containing resin was obtained after filtration and drying.

*Synthesis of MnP-MnO_2_/C, MnP/C, and MnO_2_/C:* The 3:1 Mn^2+^-containing resins and KOH were well ground and then heat-treated in a tube furnace. The heating temperature is 1175 °C, the heating rate is 5 ℃ min^−1^, and the temperature is reduced to 700 °C at a cooling rate of 5 ℃ min^−1^ after 1 h. The O_2_ is introduced for 15 min, and then it is reduced to room temperature in a N_2_ atmosphere. The heat-treated samples were washed to neutrality with deionised water and then the samples were dried to obtain MnP-MnO_2_/C. MnP/C is synthesized via the same method except the ratio of Mn^2+^-containing resin to KOH is 1:0.5 and under a N_2_ atmosphere. The MnO_2_/C obtained via cooling in an O_2_ atmosphere. The contents of MnP-MnO_2_, MnP, and MnO_2_ in the prepared compounds were 52.1 wt%, 52.4 wt%, and 52.9 wt%, respectively. The MnP content in MnP-MnO_2_/C is 26.8 wt%.

**Preparation of** **Cathodes**

*Preparation of MnP-MnO_2_/C@S, MnP/C@S, and MnO_2_/C@S Cathodes:* Typically, MnP-MnO_2_/C and sulfur powder were mixed with a weight ratio of 1:3, and then, the mixture was heated at 155 °C for 12 h. The sulfur-loaded sample was uniformly mixed with polyvinylidene fluoride (PVDF) powderand conductive carbon black (Super P) at a ratio of 8:1:1. The mixed powder was placed in N-methyl-2-pyrrolidinone (NMP) solution and stirred evenly to form a slurry. The slurry was coated on the aluminium foil, then vacuum dried at 50 °C for 12 h, and finally cut into an electrode sheet with a diameter of 14 mm with a sulfur loading of 1.8 mg cm^−2^, labeled as MnP-MnO_2_/C@S cathode. MnP/C@S and MnO_2_/C@S cathodes were prepared in the same way.

*Preparation of MnP-MnO_2_/C@S_(3-cycle)_ Cathode:* The as-assembled Li–S cell with MnP-MnO_2_/C@S cathode was galvanostatically cycled for 3 cycles. The potential range was 2.2–2.4 V and the current was 0.112 mA. Then, the working electrode was taken out from the dissembled cell, washed by tetraglyme, and dried in an Ar-filled glove box. The as-prepared electrode was denoted as MnP-MnO_2_/C@S_(3-cycle)_.

**Physical Characterization**

Powder X-ray diffraction (XRD) was collected on a D/Max-III X-ray (Rigaku Co. Ltd., Japan) with CuK-α irradiation at 40 kV and 30 mA. Scanning electron microscopy (SEM) and transmission electron microscopy (TEM) images were obtained using a sigma 300 microscope (Carl Zeiss Co. Ltd., Germany) and Tian ETEM G2 80-300 microscope (FEI Co. Ltd., USA). The Brunauer-Emmett-Teller (BET) specific surface area and pore size distribution were analyzed using an ASAP 2460 specific surface area analyzer (Micromeritics Co. Ltd., USA). UV-vis spectrophotometer (PerkinElmer Lambda 650, USA) is used to analyze the absorption spectrum after Li_2_S_6_ adsorption. X-ray photoelectron spectroscopy (XPS) was performed using an ESCALAB 250 energy spectrometer with a monochromatic Al Kα radiation source. Thermogravimetric analyzer (TGA; Netzsch STA449 F5 Jupiter) was used to determine the content of sulfur under N_2_ conditions at 30−850 °C.

**Li_2_S_6_ Symmetrical Cell Tests**

0.5 M Li_2_S_6_ solution was synthesized by mixing S_8_ and lithium sulfide (Li_2_S) in a stoichiometric molar ratio (5:1) in 1,2-dimethoxyethane/1,3-dioxolane (DME/DOL, 1:1 (v/v)). This was stirred vigorously for 24 h at a temperature of 60 °C under an Ar atmosphere. MnP-MnO_2_/C, MnP/C, and MnO_2_/C and PVDF were mixed homogeneously at a mass ratio of 9:1, and then NMP was added to make a slurry of moderate consistency, which was uniformly coated on the carbon cloth. After vacuum drying, two electrode sheets with similar mass were used as cathode and anode, and 25 μL of Li_2_S_6_ solution was added on both sides of the cathode and anode as electrolyte, and finally encapsulated into a coin cell in an Ar-shielded glove box (H_2_O < 0.1 ppm, O_2_ < 0.1 ppm). The sweep rate of 5 mV s^−1^ and the voltage range was −0.8−0.8 V.

**Linear Sweep Voltammetry (LSV) Tests**

The system consisted of a glassy carbon electrode as the working electrode, and two lithium foils as the counter and reference electrodes, respectively. The working electrode is prepared by dispersing 5 mg of MnP-MnO_2_/C sample in a mixed solution consisting of 1 mL of isopropanol and 50 μL of Nafion with a mass fraction of 0.5 wt%. After ultrasonication for 30 min, it was dropped on a glassy carbon electrode with an area of 0.196 cm^−2^. The load of the electrocatalyst on the working electrode is approximately 0.1 mg cm^−2^. The lithium foil was cut into a size of 1.5×2.0 cm^−2^ as a counter electrode and a reference electrode. The electrolyte solvent used is a mixture of DOL:DME (1:1 (v/v)) dissolved with 4 mM S_8_ and 1.0 M LiTFSI. During the whole test process, the working, counter, and reference electrodes were kept below the electrolyte level, the scanning range was 3–1.5 V, the scanning speed was 20 mV s^−1^.The working electrode is operating at a high rotational speed of 1000 rpm. The exchange current density (*J_0_*) referring to the reaction rate at zero overpotential, and can be concluded according to the Tafel equation:^[1]^

$\eta_{1}=\eta\times log(\frac{J}{J_{0}})$ (1)

Where *η_1_*, *η*, *J*, and *J_0_* are the overpotential, Tafel slope, measured current density and exchange current density, respectively.

**Li_2_S Nucleation and Dissolution Tests**

0.4 M Li_2_S_8_ solution was obtained by stirring Li_2_S and S (1:7 molar ratio) in a mixed solvent of DME/DOL (1:1 v/v) containing 1.0 M lithium (bis(trifluoromethanesulfonyl)imide) (LiTFSI) for 48 h at 60 ℃ under an Ar atmosphere. The Li_2_S nucleation tests were performed with MnP-MnO_2_/C, MnP/C, and MnO_2_/C as the electrodes, and lithium sheets as the anodes. The coin cells were assembled by adding 20 μL of Li_2_S_8_ solution on the cathode side and 20 μL of normal Li−S battery electrolyte on the anode side. The batteries were discharged to 2.06 V at a constant current of 0.112 mA and maintained at 2.05 V. The cell assembly process for Li_2_S dissolution tests is the same as that of the Li_2_S nucleation test mentioned above. The cell was first discharged to 1.8 V at a constant current (0.1 mA), and then discharged to 1.70 V at a current of 0.02 mA, confirming that Li_2_S_4_ was completely converted to Li_2_S. Finally, the cell was discharged at a constant potential of 2.35 V for 20 h to ensure the complete dissolution of Li_2_S.

**Li–S Coin and Pouch Cells Assembly and Electrochemical Performance Tests**

Coin cells were assembled with MnP-MnO_2_/C@S, MnP/C@S, MnO_2_/C@S, and C@S cathodes, lithium anodes, and electrolyte (1 M LiTFSI in DOL/DME (1:1 (v/v)) with 2 wt% LiNO_3_) in an Ar-shielded glove box. The electrolyte/sulfur (E/S) ratio was 20:1. Pouch cells were assembled using MnP-MnO_2_/C@S cathodes with the size is controlled at 5.5 × 4.5 cm^2^, and the sulfur loading of the cathode is 7.1 mg cm^−2^ for each single side. The E/S ratio is 2.5 mg mg_s_^-1^. The galvanostatic intermittent titration technique (GITT) tests of coin cells were measured at a current pulse of 12 min and the time of the following relaxation step was 30 min. The cyclic voltammetric (CV) of coin cells was measured at a scan rate of 0.1–0.5 mV s^−1^ and voltages in the range of 1.7–2.8 V. The electrochemical impedance spectroscopy (EIS) tests of coin cells were conducted using an IM6 electrochemical workstation (Zahner-Elektrik, Germany) in a frequency range of 0.01–100 kHz and a voltage amplitude of 5 mV. The galvanostatic charge-discharge (GCD) tests of coin and pouch cells were galvanostatically cycled (1 C=1672 mAh g^-1^) within a voltage window of 1.7‒2.8 V on a CT-4008T Neware battery test system (Shenzhen Neware Battery Co., China).

The energy density of the Li−S pouch cell was calculated as follows:

$E_{g}=\frac{U\times C}{\sum m_{i}}$ (2)

where *E_g_* is the energy density. *U* is the average voltage (2.1 V), *C* is the discharge capacity, and *m_i_* is the weight of each cell component.

**In Situ Characterization Techniques**

The coin cells for in situ Raman spectroscopy tests were assembled using a 2032 perforated cathode shell with a pore size of about 6 mm. A coin cell was installed in a Raman device (Beijing Scistar Technology Co. Ltd., China) with a quartz window. The charge/discharge current is 0.2 C, the voltage is set to 1.7−2.8 V, and the situ Raman spectroscopy data is collected every 0.1 V. The wavelength range of situ Raman spectroscopy is 50–600 cm^−1^. The coin cells for in situ XRD tests were assembled using a 2032 perforated cathode shell with a pore size of about 14 mm. The assembled battery was placed in an in situ XRD device (Beijing Scistar Technology Co. Ltd., China), and the side containing the metal beryllium window was used as the XRD incident window. The charge/discharge current intensity is 0.2 C, the voltage range is 1.7−2.8 V and the *2θ* diffraction angle is 10−60°.

**Density Functional Theory (DFT) Calculations**

All DFT calculations were carried out on the Vienna Ab initio Simulation Package (VASP). The GGA calculation was performed within the Perdew-Burke-Ernzerhof (PBE) exchange-correlation potential. DFT+D3 with Becke-Johnson damping was used to describe the interaction of vander Waals forces between the layers. The cut-off energy is 500 eV, and the restriction of all atomic positions and cell parameters is lifted until the average force per atom is reduced to 0.01 eV Å^−1^. Brillouin zone integration is performed by selecting a K-point grid that satisfies ka~30 in the difference. The binding energies of S_8_ and LiPS species (Li_2_S, Li_2_S_2_, Li_2_S_4_, Li_2_S_6_, and Li_2_S_8_) adsorbed on different surfaces were calculated in MnP (111) surface and MnO_2_ (110) surface. The binding energy was calculated by the equation:

$\Delta E_{ads}=E(slab+ads)-E(slab)-E(ads)$ (3)

Whereas *∆E_ads_* is the difference in DFT energy before and after adsorption, *E_slab_* and *E_ads_* are the adsorption energies of the slab and the adsorbed molecules, respectively.

The formation energy of MnS_2_ can be calculated by the following equation:

$\Delta E_{doping}=E\left( nV_{S,P} \right)-E\left( ideal \right)-N(E\left( P_{4} \right)/4-E\left( S_{8} \right)/8)$ (4)

where N is the number of S atoms substituting P atoms. All calculations were spin-polarized.

# Supporting Figures and Tables


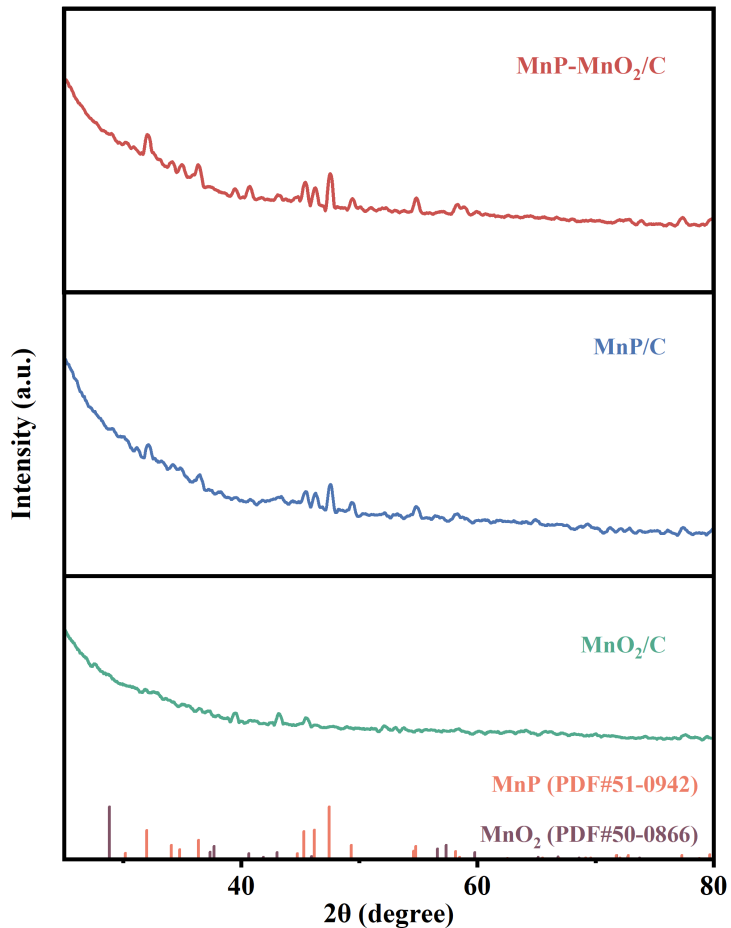


**Figure S1.** XRD patterns of MnP-MnO_2_/C, MnP/C, and MnO_2_/C.


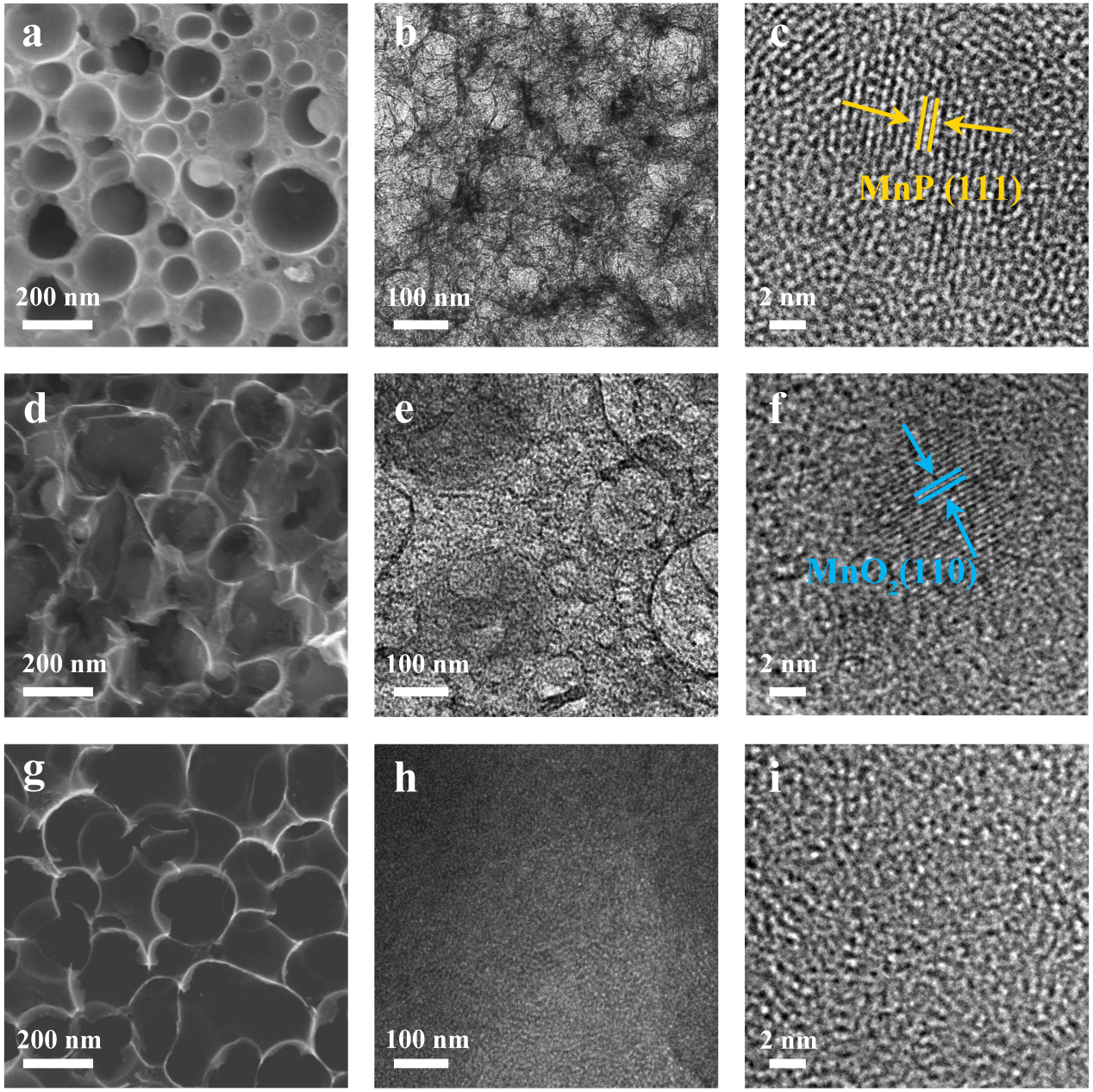


**Figure S2.** SEM, TEM, and HRTEM images of a–c) MnP/C, d–f) MnO_2_/C, and g–i) pure C.


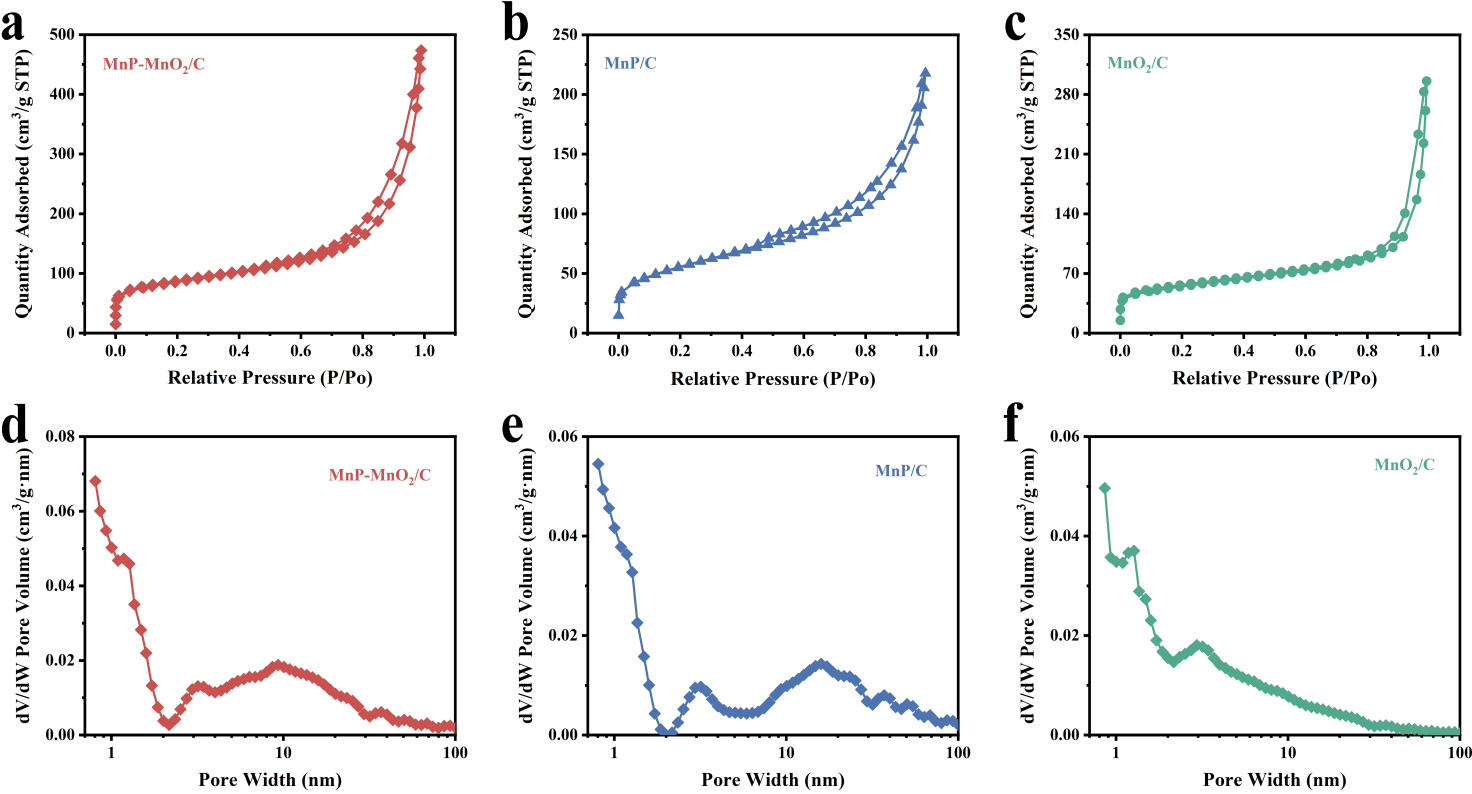


**Figure S3.** a–c) N_2_ adsorption-desorption isotherms and d–f) pore size distribution curves of MnP-MnO_2_/C, MnP/C, and MnO_2_/C.


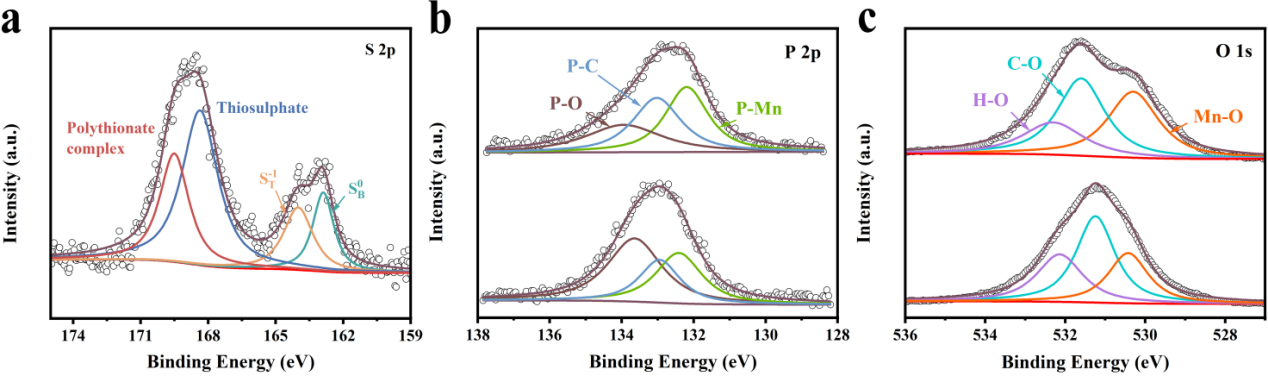


**Figure S4.** a) XPS spectra of S 2p of MnP-MnO_2_/C after Li_2_S_6_ adsorption. b–c) XPS spectra of P 2p and O 1s for MnP-MnO_2_/C before and after Li_2_S_6_ adsorption.


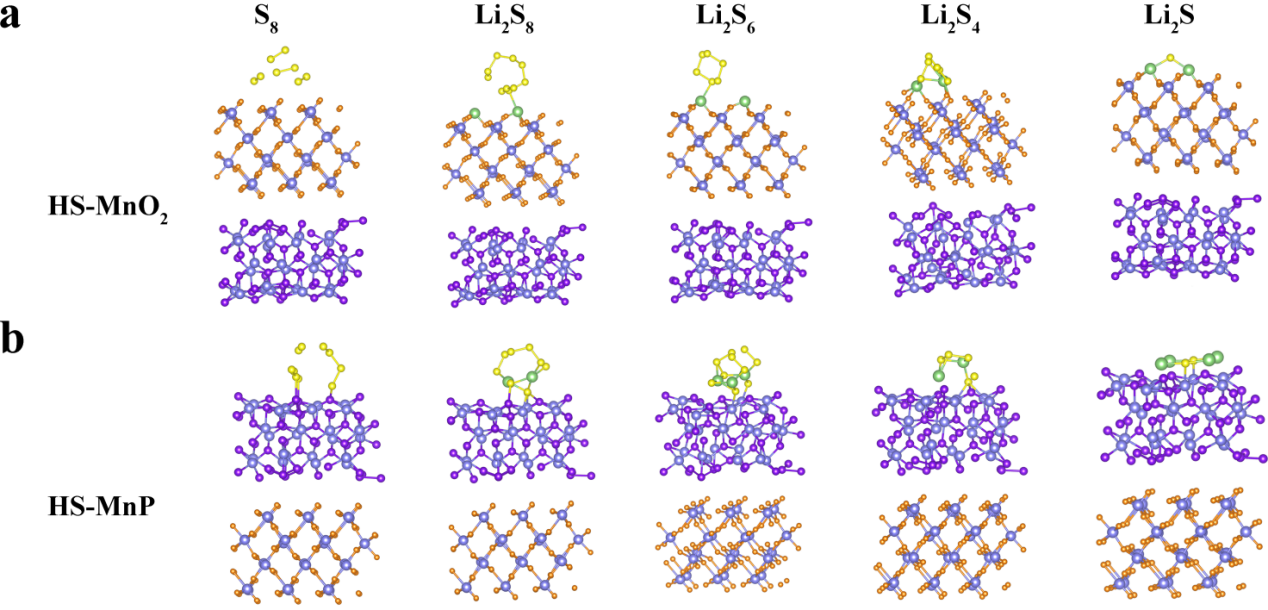


**Figure S5.** a) Optimized adsorption configurations of S_8_, Li_2_S_8_, Li_2_S_6_, Li_2_S_4_, and Li_2_S on a) the (110) planes of MnO_2_ in MnP-MnO_2_ and b) (111) planes of MnP in MnP-MnO_2_.


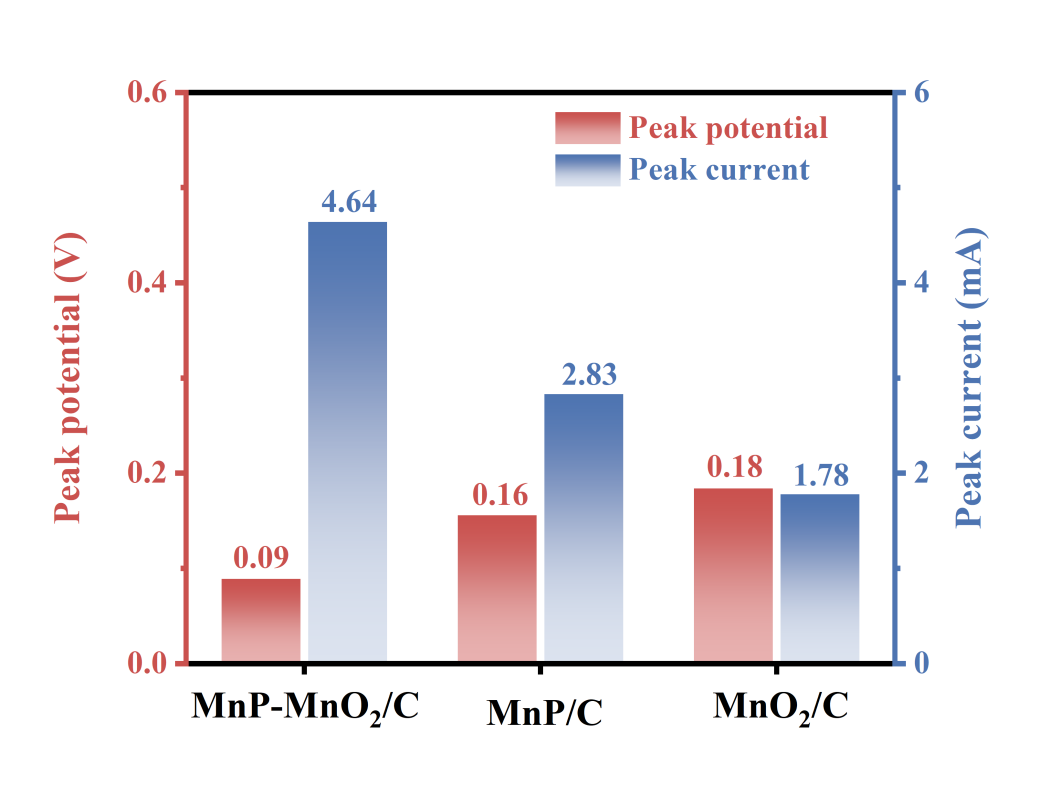


**Figure S6.** The peak potential and peak current density of peak A in CV profiles of Li_2_S_6_ symmetrical cells.


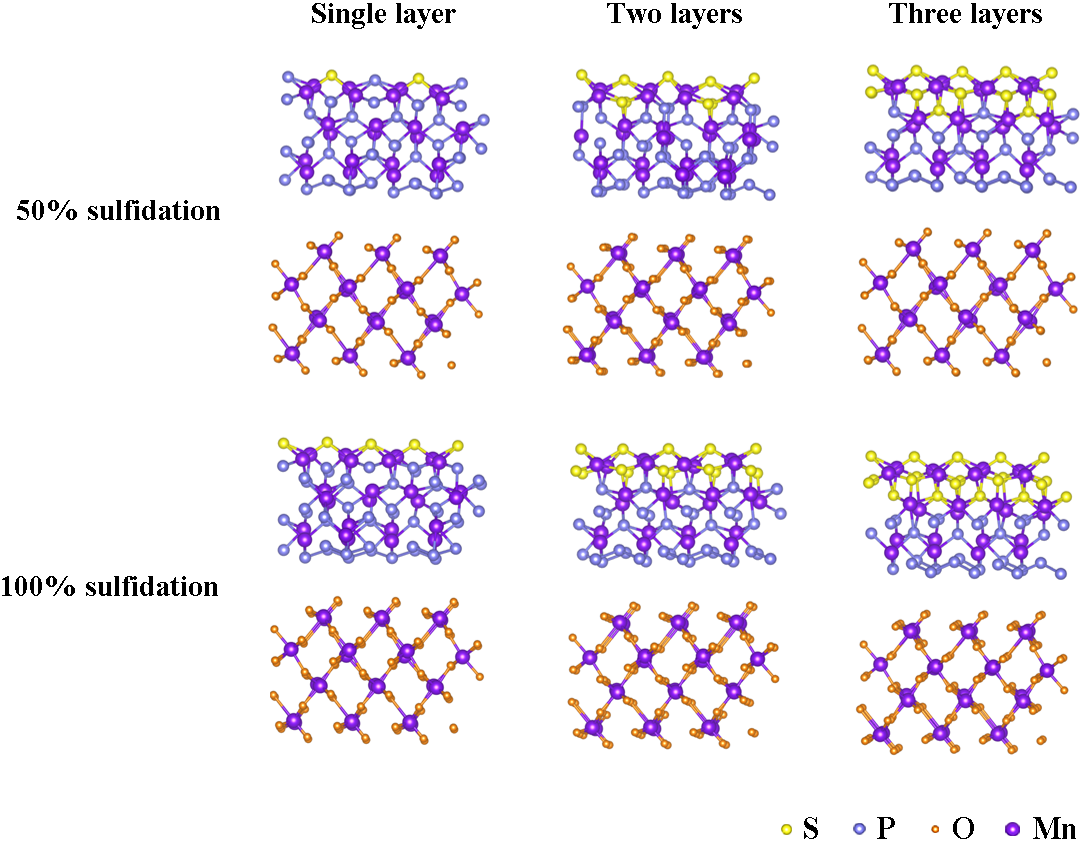


**Figure S7.** Optimized configuration after the surface sulfuration for MnP-MnO_2_ heterostructure.


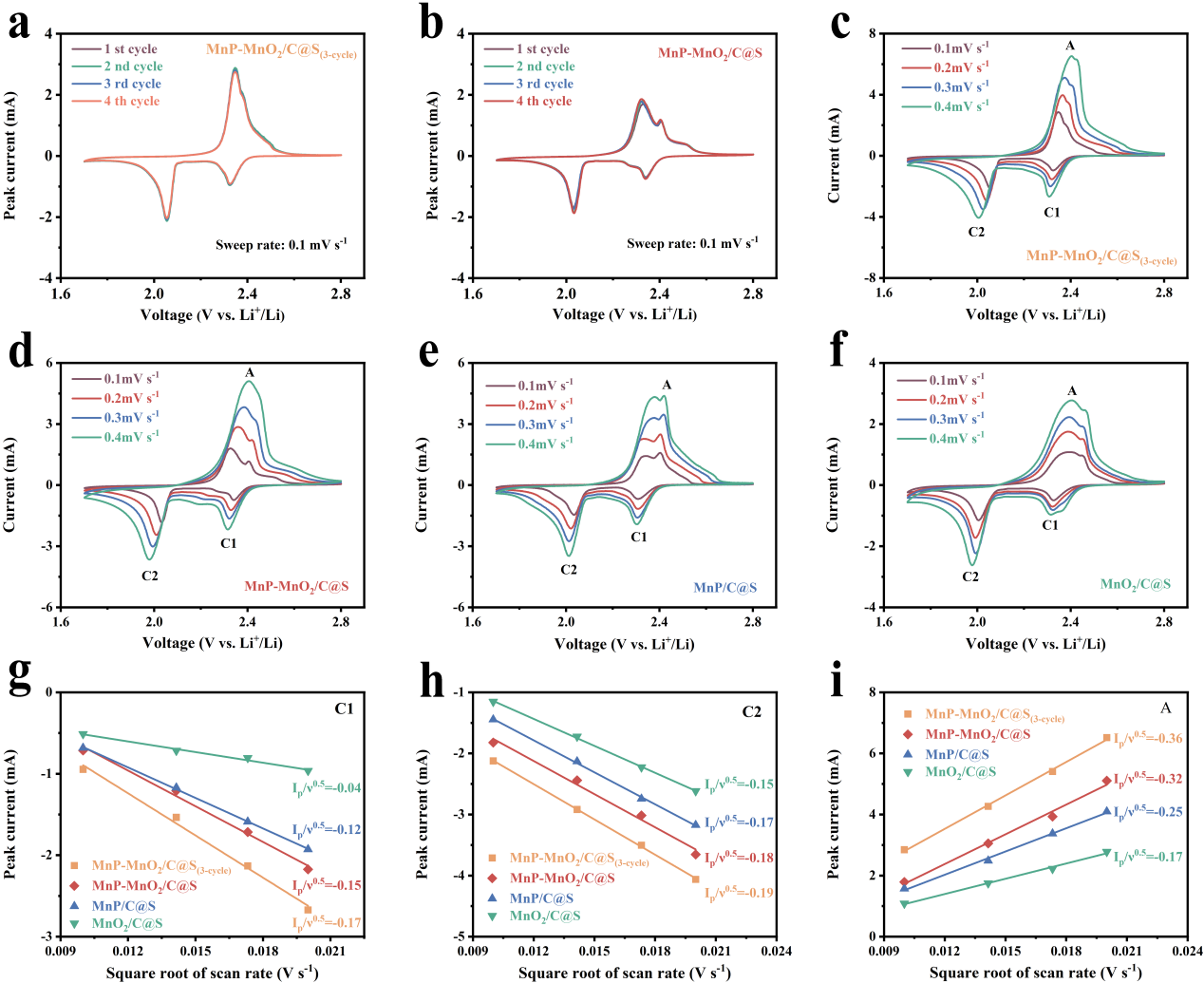


**Figure S8.** CV curves of a) MnP-MnO_2_/C@S_(3-cycle)_ and b) MnP-MnO_2_/C@S cathodes at 0.1 mV s^−1^. c−f) CV curves of MnP-MnO_2_/C@S_(3-cycle)_, MnP-MnO_2_/C@S, MnP/C@S, and MnO_2_/C@S cathodes at different sweep rates and g−i) the CV peak current for peaks C1, C2, and A versus the square root of the sweep rates.


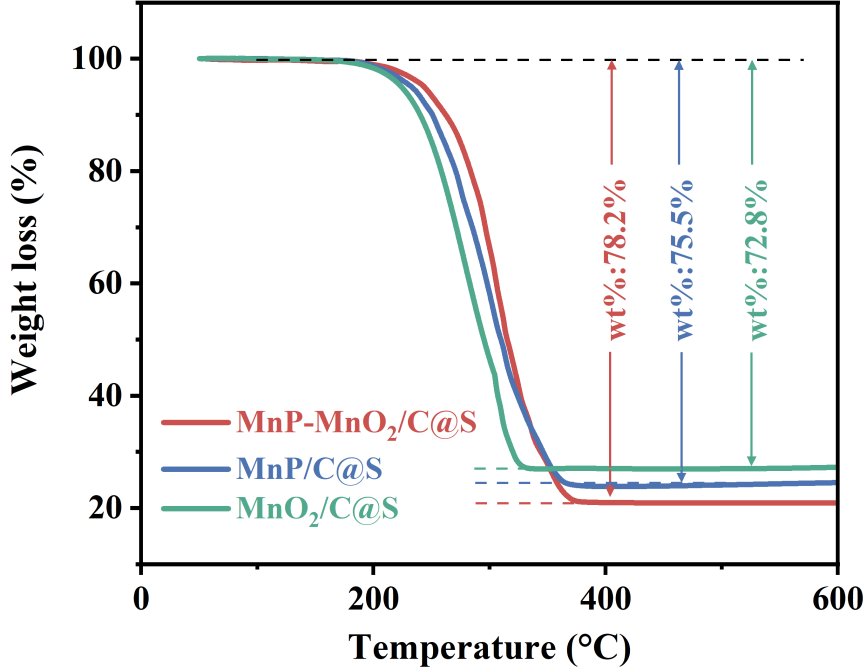


**Figure S9.** Thermogravimetric analysis (TGA) curves of MnP-MnO_2_/C@S, MnP/C@S, and MnO_2_/C@S cathodes.


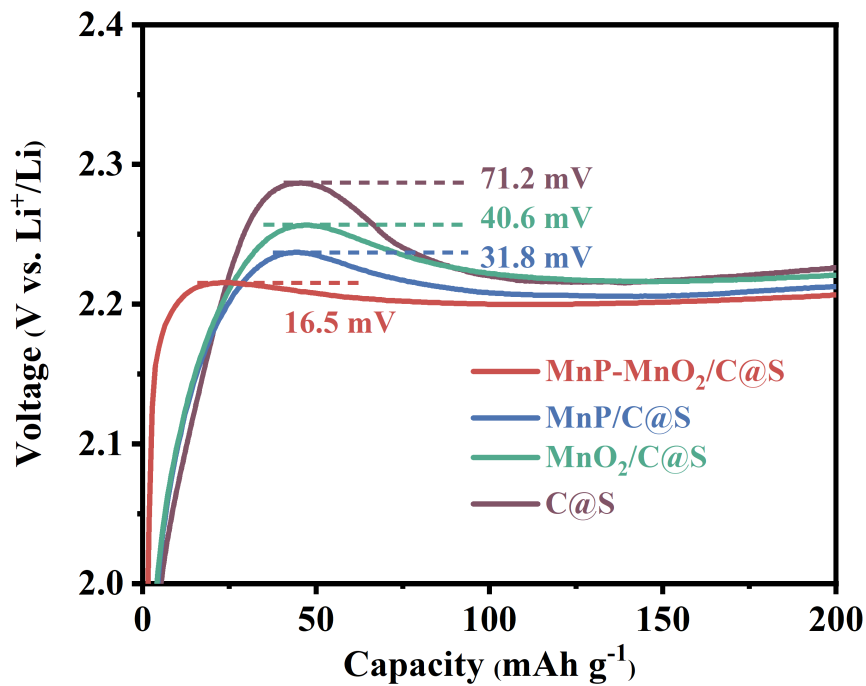


**Figure S10.** The first charge voltage profiles of MnP-MnO_2_/C@S, MnP/C@S, MnO_2_/C@S, and C@S cathodes.


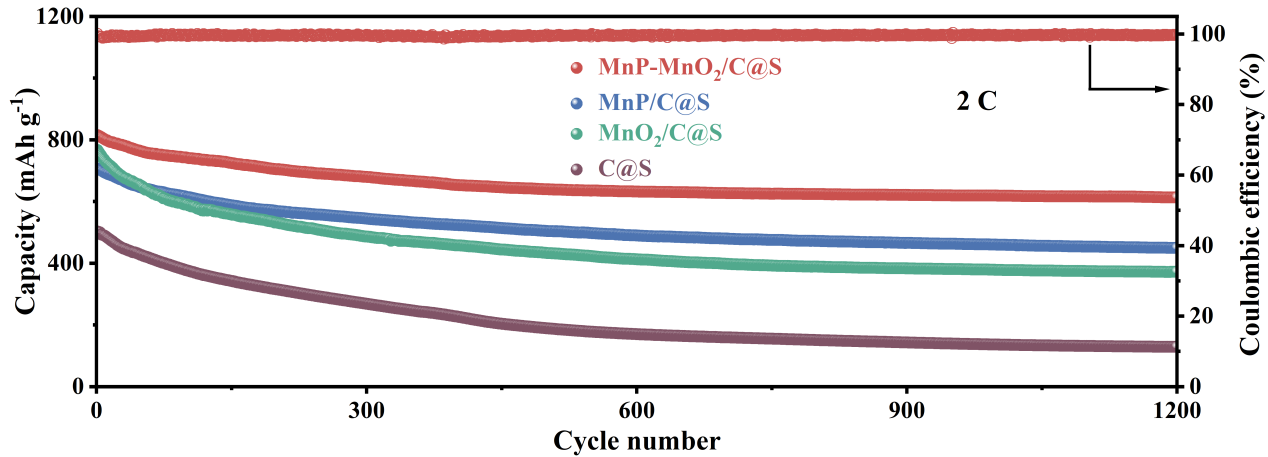


**Figure S11.** Long-term cycling performance at 2 C of MnP-MnO_2_/C@S, MnP/C@S, MnO_2_/C@S, and C@S cathodes.


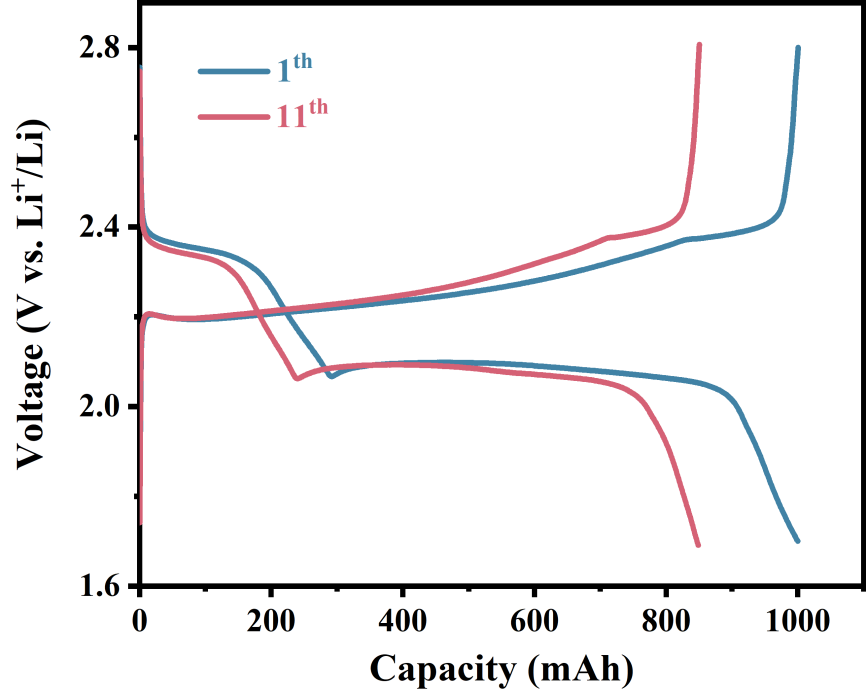


**Figure S12.** Voltage–capacity profiles of Li–S pouch cell at the 1^th^, and 11^th^ cycle.

**Table S1.** The potential difference (*Δ*) between peak C2 and peak A from the CV curves.

| **Cathodes** | **Peak C2 potential (V)** | **Peak A potential (V)** | ***Δ* (mV)** |
| --- | --- | --- | --- |
| MnP-MnO_2_/C@S_(3-cycle)_ | 2.056 | 2.338 | 282 |
| MnP-MnO_2_/C@S | 2.032 | 2.327 | 294 |
| MnP/C@S | 2.033 | 2.403 | 370 |
| MnO_2_/C@S | 2.004 | 2.414 | 410 |

**Table S2.** Impedance of EIS for different cathode materials.

| **Cathodes** | ***R_internal_* (Ω)** | ***R_ct_* (Ω)** |
| --- | --- | --- |
| MnP-MnO_2_/C@S_(3-cycle)_ | 2.02 | 12.20 |
| MnP-MnO_2_/C@S | 2.53 | 16.64 |
| MnP/C@S | 2.90 | 33.65 |
| MnO_2_/C@S | 3.11 | 43.54 |

**Table S3.** Performance comparison of the MnP-MnO_2_/C catalyst with the recent reported works.

| **Material** | **Sulfur loading**  **(mg cm**^−2^) | **Initial specific capacity (mAh g**^−1^) | **Rate (C)** | **Cycle**  **number** | **Capacity**  **decay rate (%)** |
| --- | --- | --- | --- | --- | --- |
| **MnP-MnO_2_/C**  **(This work)** | **1.8** | **815** | **2** | **1200** | **0.021** |
|  |  | **656.5** | **5** | **2000** | **0.017** |
| Na_0.67_Ni_0.25_Mn_0.75_O_2_-MnS_2_-Ni_3_S_4_^[2]^ | 1.0 | 1040 | 2 | 400 | 0.070 |
| Mn_3_O_4−x_^[3]^ | 1.5 | 572 | 2.5 | 2000 | 0.028 |
| Mn_3_O_4_-MnS^[4]^ | 2.0 | 1074 | 2 | 1000 | 0.016 |
| MnS-MoS_2_^[5]^ | 1.4 | 778 | 2 | 1000 | 0.048 |
| CoNiO_2_/Co_4_N-G^[6]^ | 1 | 688 | 4 | 1100 | 0.056 |
| MoS_2_–MoN^[7]^ | 1.2 | 778.0 | 2 | 2000 | 0.041 |
| CNTs@TiN–TiO_2_^[8]^ | 3.3 | 900 | 2 | 500 | 0.031 |
| Mn_3_O_4_−MnP_x_^[9]^ | 1.5 | 810 | 2 | 1000 | 0.043 |
| Co_9_S_8_/CoO-G^[10]^ | 1.0 | 925 | 1 | 1000 | 0.065 |
| Co/CoS_2_@NSC^[11]^ | 1.0 | 1054.4 | 1 | 500 | 0.072 |
| v-ZnTe/CoTe_2_@NC^[12]^ | 1.5 | 900 | 1 | 500 | 0.022 |

References

[1] L. Peng, Z. Wei, C. Wan, J. Li, Z. Chen, D. Zhu, D. Baumann, H. Liu, C.S. Allen, X. Xu, A.I. Kirkland, I. Shakir, Z. Almutairi, S. Tolbert, B. Dunn, Y. Huang, P. Sautet, X. Duan, Nat. Catal. **2020**, 3, 762.

[2] P. Zeng, H. Zou, C. Cheng, L. Wang, C. Yuan, G. Liu, J. Mao, T.S. Chan, Q. Wang and L. Zhang, Adv. Funct. Mater. **2023**, 33, 2214770.

[3] Y. Zhu, Y. Zuo, X. Jiao, R. Manjunatha, E.R. Ezeigwe, W. Yan and J. Zhang, Carbon Energy **2022**, 5, e249.

[4] B. Qin, Q. Wang, W. Yao, Y. Cai, Y. Chen, P. Wang, Y. Zou, X. Zheng, J. Cao, J. Qi and W. Cai, Energy Environ. Mater. **2023**, https://doi.org/10.1002/eem2.12475e12475.

[5] W. Xiong, J. Lin, H. Wang, S. Li, J. Wang, Y. Mao, X. Zhan, D.-Y. Wu and L. Zhang, J. Energy Chem. **2023**, 81, 492.

[6] J. Pu, W. Gong, Z. Shen, L. Wang, Y. Yao and G. Hong, Adv. Sci. **2022**, 9, e2104375.

[7] S. Wang, S. Feng, J. Liang, Q. Su, F. Zhao, H. Song, M. Zheng, Q. Sun, Z. Song, X. Jia, J. Yang, Y. Li, J. Liao, R. Li and X. Sun, Adv. Energy Mater. **2021**, 11, 2003314.

[8] H. Zhang, L.K. Ono, G. Tong, Y. Liu and Y. Qi, Nat. Commun. **2021**, 12, 4738.

[9] K. Guo, G. Qu, J. Li, H. Xia, W. Yan, J. Fu, P. Yuan and J. Zhang, Energy Storage Mater. **2021**, 36, 496.

[10] N. Wang, B. Chen, K. Qin, E. Liu, C. Shi, C. He and N. Zhao, Nano Energy **2019**, 60, 332.

[11] M. Zheng, J. Zhao, W. Wu, R. Chen, S. Chen and N. Cheng, Small **2023**, https://doi.org/10.1002/smll.202303192e2303192.

[12] C. Huang, J. Yu, C. Li, Z. Cui, C. Zhang, C. Zhang, B. Nan, J. Li, J. Arbiol and A. Cabot, Adv. Funct. Mater. **2023**, 33, 2305624.
